# Establishing next-generation pest control services in rice fields: eco-agriculture

**DOI:** 10.1038/s41598-019-46688-6

**Published:** 2019-07-15

**Authors:** M. P. Ali, M. N. Bari, S. S. Haque, M. M. M. Kabir, S. Afrin, F. Nowrin, M. S. Islam, D. A. Landis

**Affiliations:** 1 Entomology Division, Bangladesh Rice Research Institute, Gazipur, 1701 Bangladesh; 2 Farm Management Division, Bangladesh Rice Research Institute, Gazipur, 1701 Bangladesh; 3 Department of Entomology, Michigan State University, East Lansing, MI USA

**Keywords:** Entomology, Agroecology

## Abstract

Pesticides are commonly used in food crop production systems to control crop pests and diseases and ensure maximum yield with high market value. However, the accumulation of these chemical inputs in crop fields increases risks to biodiversity and human health. In addition, people are increasingly seeking foods in which pesticide residues are low or absent and that have been produced in a sustainable fashion. More than half of the world’s human population is dependent on rice as a staple food and chemical pesticides to control pests is the dominant paradigm in rice production. In contrast, the use of natural enemies to suppress crop pests has the potential to reduce chemical pesticide inputs in rice production systems. Currently, predators and parasitoids often do not persist in rice production landscapes due to the absence of shelter or nutritional sources. In this study, we modified the existing rice landscape through an eco-engineering technique that aims to increase natural biocontrol agents for crop protection. In this system, planting nectar-rich flowering plants on rice bunds provides food and shelter to enhance biocontrol agent activity and reduce pest numbers, while maintaining grain yield. The abundance of predators and parasitoids and parasitism rates increased significantly in the eco-engineering plots compared to the insecticide-treated and control plots. Moreover, a significantly lower number of principal insect pests and damage symptoms were found in treatments where flowering plants were grown on bunds than in plots where such plants were not grown. This study indicates that manipulating habitat for natural enemies in rice landscapes enhances pest suppression and maintains equal yields while reducing the need for insecticide use in crop fields.

## Introduction

Rice (*Oryza sativa*) is the most important staple food for a large part of the world’s human population, especially in East and South Asia, the Middle East, Latin America, and the West Indies^1^.Globally, rice provides more than one-fifth of the calories consumed by humans. Outbreaks of rice-feeding insect pests are thus a serious threat to food security. Recently, rice yield losses increased due to widespread outbreaks of the brown plant hopper (*Nilaparvata lugens*)^2,3^, rice leaffolder (*Cnaphalocrocis medinalis* Güenée)^4^, small brown planthopper (*Laodelphax striatellus* Fallen), rice hispa (*Dicladispa armigera* Oliver), yellow stem borer (*Scirpophaga incertulas* L.) and white-backed planthopper (*Sogatella furcifera* Horvath). These pests cause hundreds of millions of dollars of losses every year and threaten food security in regions where rice is the staple food^5^. Recent studies^6^ have shown that insect pest outbreaks can be traced to the misuse of insecticides^7^ threatening the entire rice ecosystem^8^.

Among the key pest of rice, planthoppers (Hemiptera: Delphacidae) directly damage plants by sucking the cell sap from the base (stem) of the plants^9^. Their feeding also transmits viruses, including rice ragged stunt (RRSV) and rice grassy stunt (RGSV)^10,11^, which caused the loss of 828,000 tons of rice valued at US$120 million in southern Vietnam in 2005–2006^12^. Research at Bangladesh Rice Research (BRRI) and the International Rice Research Institute (IRRI) has demonstrated the high costs of planthopper outbreaks on rice plantations^6,13–15^. Outbreaks of planthoppers have been linked to the increased use of broad-spectrum insecticides^16–24^ which cause adverse effects on natural enemies and thus have a negative impact on rice production. In contrast, improved management of rice ecosystems can enhance natural enemies which provide natural control of pest populations^25^. Enhancing existing biocontrol agents can also reduce the need for augmenting natural enemies via mass release, reducing expense and risks associated with importing foreign species^26–29^.

Conservation biological control involves modifying the environment or existing practices to protect and enhance natural enemies and reduce pest damage^30^. Habitat management is a form of ecological engineering aimed at providing food and shelter for natural pest control agents. Carefully planned habitat manipulations can increase the abundance and effectiveness of natural enemies while promoting biodiversity and structural complexity of agroecosystems^31,32^. In particular, providing natural enemies with needed resources such as nectar^33^, pollen^34^, physical refugia^35^, alternative prey^36^, alternative hosts^37^ and lekking sites^38^.

**Figure 1:**
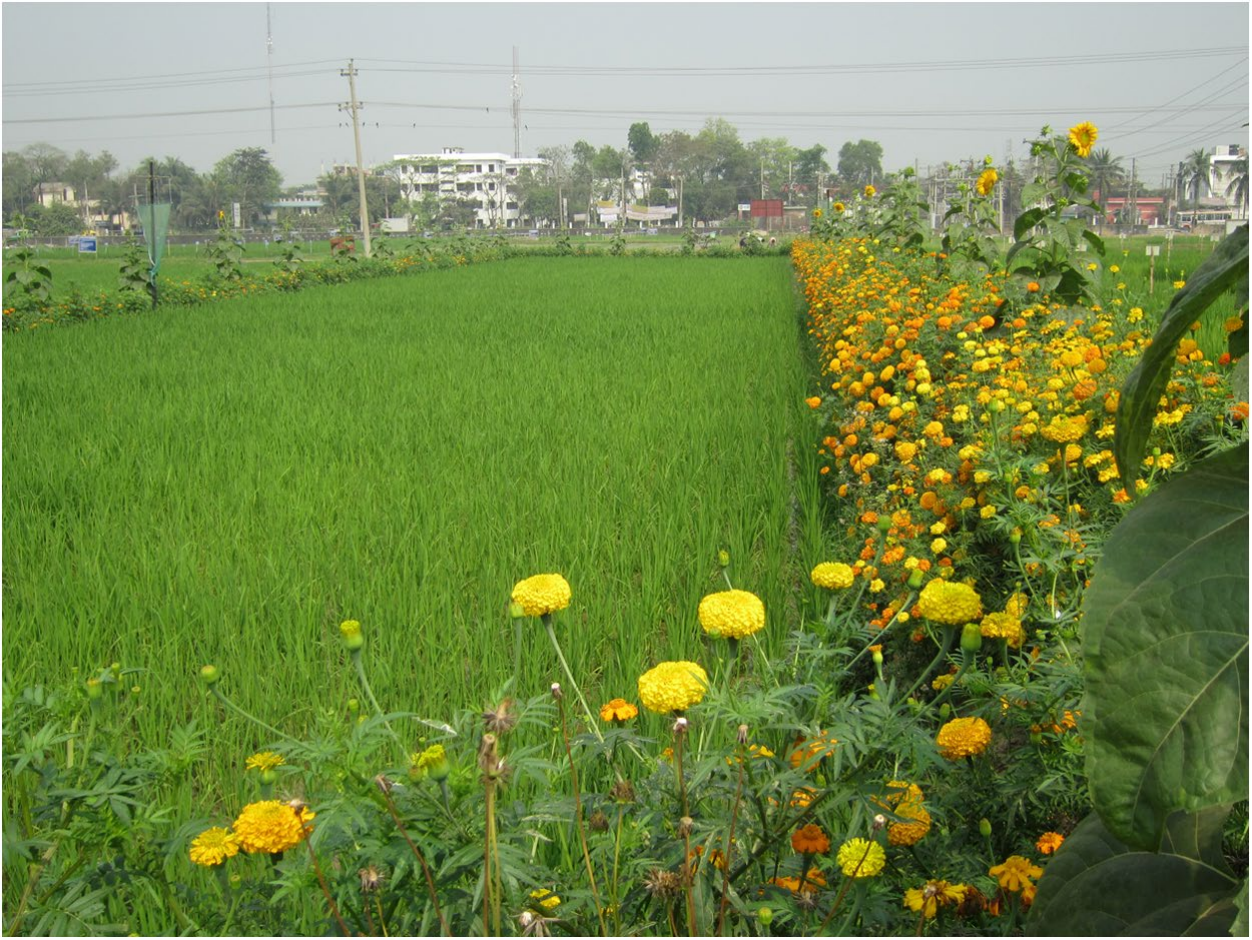
Flowering plants (sunflower, marigold, cosmos) grown on the bunds in rice plots to provide resources for biocontrol agents, especially parasites/parasitoids, in rice landscapes during Boro 2017–18, BRRI, Gazipur.

The concept of ecological engineering has been used to restore or enhance biodiversity in the rice landscape^39–42^. Unlike many flowering plants, rice lacks floral or extra-floral nectar resources that can be used by natural enemies. Planting additional nectar-rich flowering crops in rice landscapes can enhance year-round resource for natural enemies. In Bangladesh, individual rice fields are typically surrounded by an earthen ridge approximately 0.5 m in height to retain irrigation water. This ridge is commonly known as a rice bund or ail and is typically fallow throughout the year. Under eco-agricultural management, bunds are enriched with nectar producing plants and non-rice habitats used to grow perennial plants to provide additional food and shelter for natural enemies^43,44^. These improved habitat-characteristics can greatly influence natural enemy longevity, fecundity and behavior and lead to reduced pest abundance^42,45–48^. The main goal of this study was to test the efficacy of an eco-agricultural system in promoting natural enemies, reducing crop pests and maintaining rice yield. We contrasted three treatments; growing nectar-rich plants (marigold, cosmos, sunflower and sesame) on bunds (Fig. 1) coupled with no insecticides on rice (hereafter T_1_), compared to fallow bunds with (T_2_), and without prophylactic insecticide use on rice (T_3_).

## Results

### Gazipur

**Figure 2:**
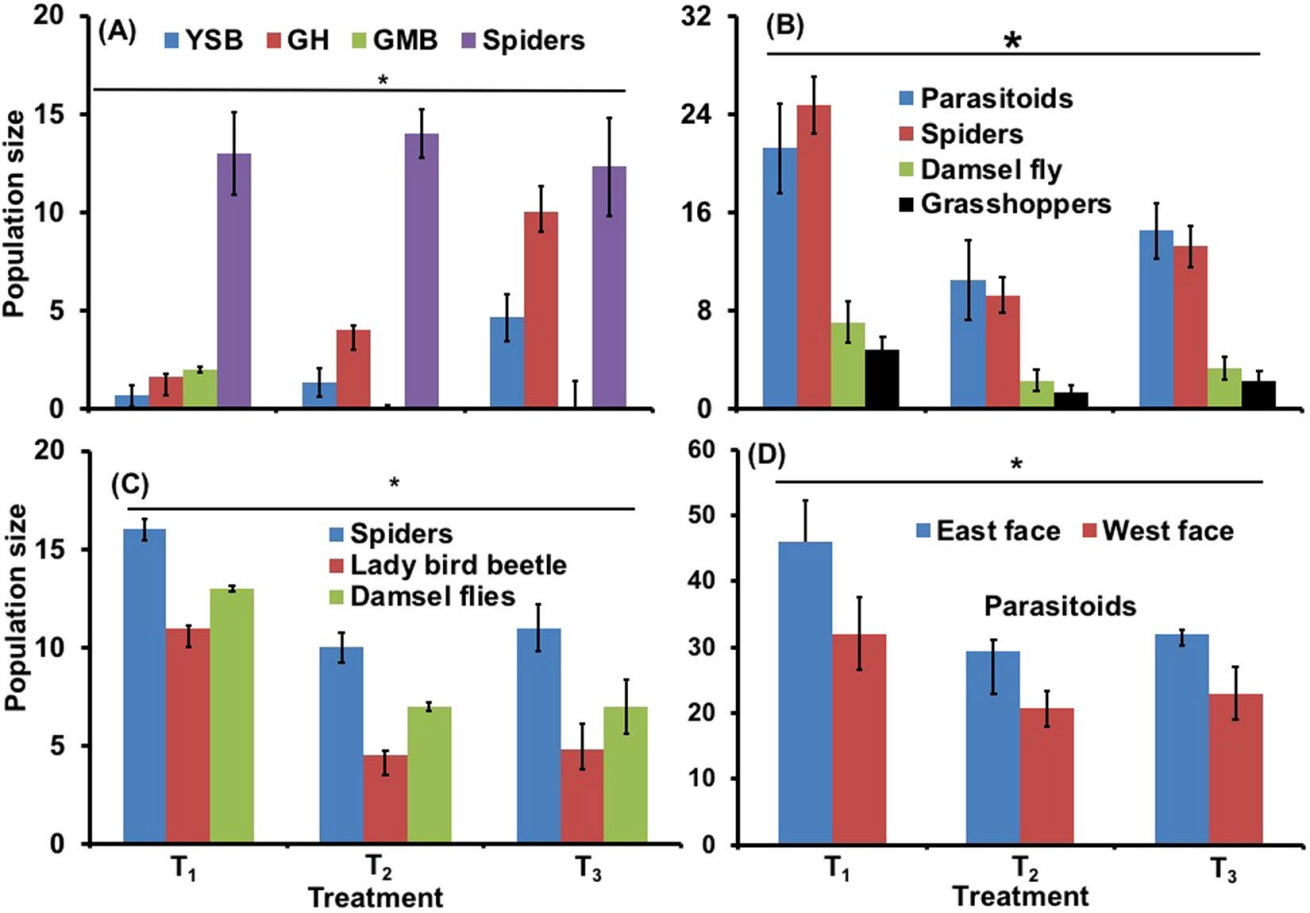
Impacts of treatments on the abundance of insect pests and natural enemies in a rice field at Gazipur. (**A**) Boro 2015–16; (**B**) Boro 2016–17; (**C**) Boro 2017–18; (**D**) parasitoid captured using yellow sticky traps during Boro 2016–17. T_1_ = Flowering plants grown on rice bunds, T_2_ = Prophylactic insecticide use, and T_3_ = Control. *Indicates a significant difference among treatments at the 5% level of significance. Error bar indicates standard error. YSB = Yellow stem borer, GMB = Green mirid bug, GH = Grasshoppers.

Insect pests and natural enemies observed during the three Boro seasons (2015–16, 2016–17 and 2017–18) are presented in Fig. 2. The results show that the highest number of grasshoppers (GHs) and yellow stem borers (YSBs) were found in T_3_ during Boro 2015–16 (Fig. 2A). A significantly lower number of GHs was found in the T_1_ treatment (*P* < 0.05). Other insect pests were not observed and are not presented here. The green mirid bug (GMB) is the most important predator of brown planthoppers in rice fields. Populations of GMBs were observed in only the T_1_ treatment (Fig. 2A). In subsequent years, significantly higher numbers of natural enemies, including spiders, damsel flies, and lady bird beetles were found in T_1_ than in the other treatments (Fig. 2B,C, *P* < 0.05). We also recorded parasitoids of rice insect pests using yellow sticky traps. The highest number of parasitoids were observed in the T_1_ treatment plots during the Boro season in 2016–17 and traps facing east and west in each field caught similar numbers of parasitoids (Fig. 2D).

**Figure 3:**
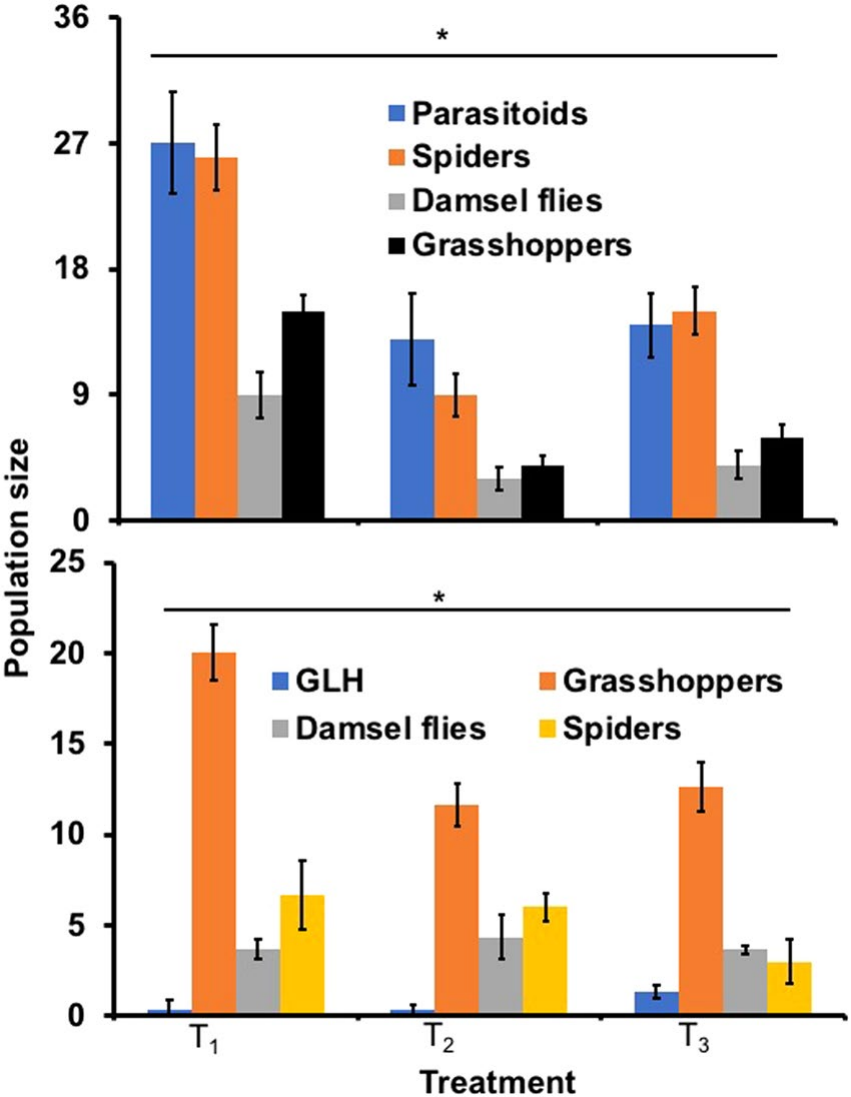
Impacts of flowering plants grown in plots on the population of arthropods at Gazipur. Upper panel represents T. Aman 2016 and lower panel represents T. Aman 2017 season. T_1_ = Flowering plants grown on rice bunds, T_2_ = Prophylactic insecticide use, and T_3_ = Control. *Indicates a significant difference among treatments at the 5% level of significance. Error bar indicates standard error.

In the T. Aman 2016 season, grasshoppers were more abundant in the T_1_ treatment, but remained below the economic threshold level (ETL) (Fig. 3, upper panel) and a similar pattern was observed in 2017 (Fig. 3, lower panel). However, grasshoppers generally do not hamper successful rice production and are seldom treated in the absence of other key pests. In 2017, abundance of a key economic insect pest, the green leafhopper (GLH) was lower in T_1_ than the control treatment (Fig. 3, lower panel). In 2016, parasitoids, spiders and damsel flies were more abundant in T_1_ (Fig. 3, upper panel) but not in 2017 (Fig. 3, lower panel).

**Figure 4:**
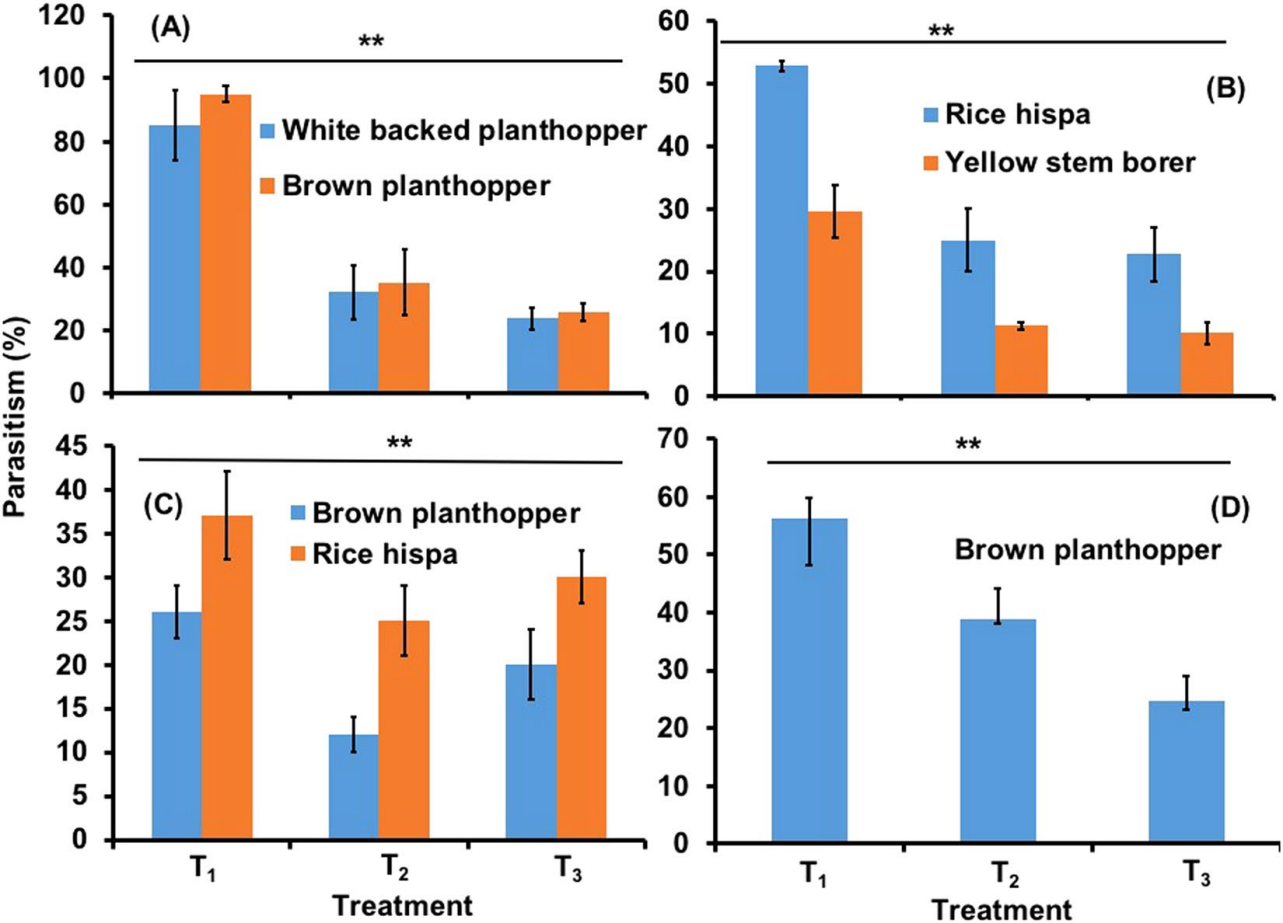
Effect of treatment on the parasitism rate of sentinel insect pest eggs in the different treatments in rice fields. T_1_ = Flowering plants grown on rice bunds, T_2_ = Prophylactic insecticide use, and T_3_ = Control (no insecticide and no flowering plants used). (**A**) T. Aman 2016; (**B**) T. Aman 2017; (**C**) Boro 2017–18; (**D**) Parasitism rate of BPH eggs during Boro 2015–16. *Indicates a significant difference among treatments at the 5% level of significance. Error bars indicate standard errors.

Parasitism of key pests, brown planthopper (BPH), white-backed planthopper (WBPH), rice hispa (RH) and yellow stem borer (YSB) during T. Aman 2016 and 2017 and Boro 2015–16 and 2017–18 also differed among treatments (Fig. 4). In all cases parasitism of pest eggs was significantly higher in T_1_ than in other treatments (Fig. 4, *P* < 0.01). Parasitism of planthopper eggs was higher in the T. Aman season, exceeding 80% (Fig. 4A) than in the Boro season when it never exceeded 60% (Fig. 4C,D).

**Figure 5:**
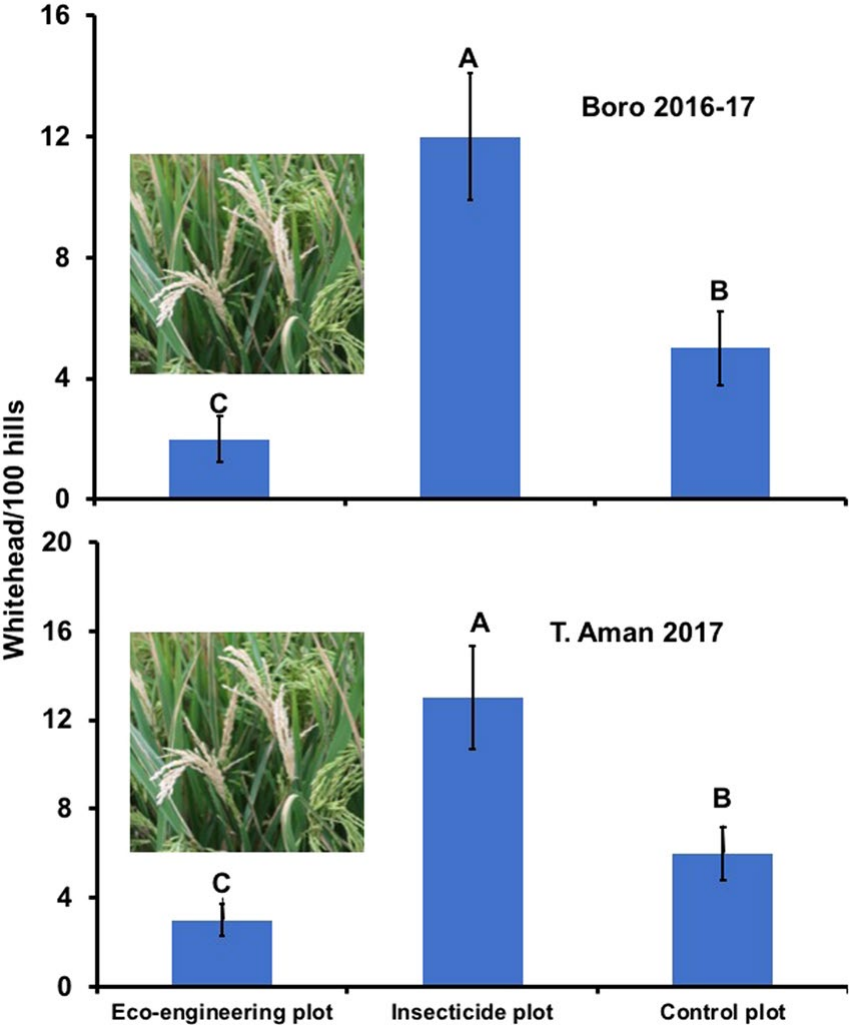
Effects of treatments on the damage symptom (white head) due to stem borer infestation in a rice field at the BRRI, Gazipur. Data were collected from 100 hills randomly selected from each experimental plot, and the mean values are presented here. Bars bearing different letters differed significantly at the 5% level. Error bars indicate the standard error.

**Figure 6:**
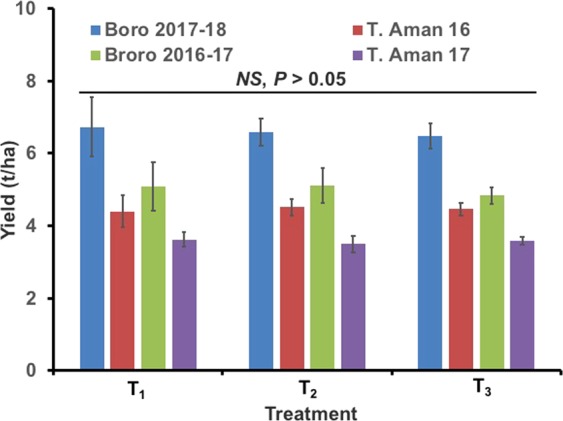
Effects of the treatments on the rice yield during the different years and seasons. BRRI dhan52 was used during T. Aman 2016 and 2017; BRRI dhan58 and BRRI dhan28 were used during Boro 2016–17 and Boro 2017–18 respectively. T_1_ = Flowering plants grown on rice bunds, T_2_ = Prophylactic insecticide use, and T_3_ = Control. *NS* indicates nonsignificant at the 5% level of significance. Error bars indicate standard errors.

The presence of flowering plants on rice bunds had a significant impact on the damage intensity caused by YSBs (P = 0.05). The highest incidence of white head per 100 hills due to YSB infestation was found in T_2_ plots, where insecticide was applied three times during the rice growing period (Fig. 5). The lowest incidence of white head was in the T_1_ plots, which were not sprayed with insecticide and bordered by sesame/marigold/cosmos. No significant impact of any treatment on yield per hectare of land was observed (Fig. 6) (P > 0.05).

### Rajshahi

Results for pest and natural enemy abundance as well as yield impacts were broadly similar at the Rajshai site. During Boro 2015–16, the highest number of grasshoppers (GHs), white leafhoppers (WLHs) and rice bugs (RBs) were found in T_1_ (Fig. S1). However, their incidence was below the ETL. Significantly higher numbers of spiders, damsel flies, lady bird beetles and carabid beetles were also found in T_1_ (Fig. S1, *P* < 0.05). In T_3_, insecticide was applied four times, none the less, yields were similar to that of T_1_ and T_2_. In T. Aman 2015, significantly higher number of YSBs and white leafhopper (WLHs) were observed in the T_2_ treatment (Fig. S2, *P* < 0.05). More importantly, T_1_ hosted a significantly higher number of natural enemies, including spiders, lady bird beetles, carabid beetles and damsel flies (Fig. S2), while T_3_ had significantly lower abundance of natural enemies, indicating a detrimental effect of insecticide (*P* < 0.05). Yield across the three treatments were similar (Fig. S3, *P* > 0.05). In the Boro 2016–17 season, the highest number of economic pests, YSBs, GHs, WLHs and stink bugs, were found in the T_3_ treatment (Fig. S1) while significantly higher numbers of lady bird beetles and damsel flies were found in T_1_ (Fig. S2, *P* < 0.05). Again, yields across the three treatments were similar (Fig. S3, *P* > 0.05).

## Discussion

Two hundred thirty-two insect pest species, 183 parasitoids and 192 predators are known to occur in Bangladesh rice ecosystems^49^. However, fewer than 20 species are considered significant pests capable of causing yield losses if they infest plants in sufficiently large numbers. Predators and parasitoids often attack these pests and control them naturally in the field. Over the last three decades, the introduction of high-yielding rice varieties to feed the fast-growing human population in developing countries, such as Bangladesh, has resulted in the use of large amounts of chemical insecticides. This heavy reliance on chemical insecticides reduces natural enemy populations in rice landscapes, promoting pest outbreaks^15^. In this study, we show that modification of existing rice landscapes via ecological engineering techniques in which nectar-rich flowering plants are grown on rice bunds surrounding rice fields promotes biocontrol agents, reduces pests and maintains crop yields.

In Bangladesh, land for rice production is commonly fragmented into numerous small plots held by individual farmers. Each rice plot has a bund around the plot. This bund is typically fallow and serves as a boundary to separate the plots owned by other land holders. We grew nectar-rich flowering plants, including marigold, cosmos, sunflower and sesame, on the bund of rice plots, which provided food, shelter and other essential nutrients for biocontrol agent development and reproduction in the rice landscape (Fig. 1). Parasitoids regularly consume nectar from flowers for sugars and carbohydrates, which increases their fitness (reproduction rate, population size, longevity, etc.)^50^. Our results show that adding flowering plants to rice bunds resulted in a significantly higher abundance of parasitoids and predators (Figs 2B,C, 3) and increased parasitism of planthopper, rice hispa, and yellow stem borer eggs in the rice field (Fig. 4). Parasitoids observed in the field or that emerged from parasitized eggs were identified as *Trichogramma zahiri*, *T*. *chilonis*, *Anagrus* spp., tachinid flies and many other ichneumon wasps. In previous studies, sesame flowers planted on bunds in the T. Aman season provided food and shelter resources to natural enemies, including hymenopteran parasitoids^40^. Sesame flowers have been found to significantly improve searching success for planthopper egg parasitoids of the genus *Anagrus*. Zheng *et al*.^50^ reported that adding nectar-rich plants and withholding insecticide consistently and significantly increased the rate of parasitism of planthopper eggs and maximized the observation of planthopper and lepidopteran egg parasitoids in rice fields.

In our study, adding nectar-rich flowering plants to rice bunds primarily facilitates parasitoids, which are recognized as essential pest control agents globally^51^. Several parasitoids have been identified for *C*. *suppressalis*, *C*. *medinalis*, *D*. *armigera*, *S*. *incertulas* and planthoppers. Forty-two parasitoids of *C*. *suppressalis*, 44 parasitoids of *C*. *medinalis* and 16 parasitoids of planthoppers have been identified as effective control agents^52^. *Anagrus* spp. were commonly found in our experiment, and these egg parasitoids have also been found to be effective against planthoppers in other Asian rice-growing countries^40,52^. Significantly higher spider populations were observed in the rice plot where the nectar-rich flowering plants were treated with insecticides. Spiders are considered to be very effective predators of many insects worldwide as well as in rice fields^52–54^. Nyffeler and Birkhofer^55^ reported that global spider communities might destroy 400–800 million tons of insect pest species. Tetragnathidae is the most dominant spider taxon in rice ecosystems^56^.

The lowest parasitism rate and catches of parasitoids of planthoppers, lepidopteran and coleopteran eggs and larvae were observed in the T_2_ treatment, where broad spectrum insecticides were applied three to four times in the absence of nectar-rich flowering plants (Figs 2B,C, 3, 4). Insecticide use reduced the abundance of parasitoids in the field, thus inducing a lower parasitism rate of planthopper, rice hispa and yellow stem borer eggs. Application of insecticides in rice is known to have a negative impact on planthopper natural enemies^17–24,50^. The use or misuse of insecticides can induce planthopper outbreaks in rice fields by lowering the number of parasitoids and predators^2^. Therefore, unsurprisingly the highest number of natural enemies and lowest number of insect pests were observed in the rice field near nectar-rich flowering plants that were not treated with insecticides. The lowest number of natural enemies (parasitoids, predators) and parasitism rates were found in rice fields where insecticides were used in the T_2_ treatment. In the control plot (T_3_ treatment), withholding insecticide in the absence of nectar-rich flowering plants on the bund resulted in higher parasitism rates and catches of planthopper and yellow stem borer egg parasitoids than those in the T_2_ treatment but significantly lower values than those in the T_1_ treatment (Figs 2–4). This confirms that the application of insecticides in rice fields can reduce the number of parasitoids and the parasitism rate of the tested insect pest eggs. Gurr *et al*.^42^ reported a similar result in suggested that growing nectar-rich flowering plants on rice bunds can enhanced the population size of natural enemies in rice fields.

Insect pest infestation was also observed in the T_1_ plots, but the infestation levels were low and did not exceed the ETL (Figs 2A, 3)^49^. Yellow sticky traps revealed that the eco-engineering plots harbored more parasitoids than the other treatments (Fig. 2D, *P* = 0.05). Damage symptoms (white head) due to the feeding of YSB was significantly lower in the T_1_ plots than in the other plots (T_2_ and T_3_) (Fig. 5, *P* = 0.05). The highest amount of white head was observed in the T_2_ plots, where insecticides were applied prophylactically at 15-day intervals during the crop growing period. This indicates that insecticides cannot stop YSB infestation completely and can even induce higher infestation levels likely due to adverse impacts on natural enemies. Moreover, the application of regular insecticides in rice fields did not improve yield. In plots where insecticides were applied yields were similar to plots where nectar-rich flowering plants were grown on bunds without insecticide and in control plots (Fig. 6, *P* > 0.05). However, more importantly, this method reduces the application of insecticides and ultimately the amount of pesticide load in rice fields. Our experimental results showed that modification of the existing rice landscape (via eco-engineering) promotes pest control without any yield penalty. In addition, the application of insecticides increased the cost of crop production, ultimately reducing the benefit cost ratio (BCR). The economic analysis (BCR) also confirms that the system we proposed here has an economic benefit. The highest BCR was found in the T_1_ treatment (supplementary information, Table S1 and S2). Growing flowering crops on rice bunds without insecticides increased the BCR. In addition to increasing the BCR, the T_1_ treatment most importantly improves the environmental quality and safety for humans, animals and biodiversity. Planting nectar-rich plants on bunds may also provide additional income sources to poor rice farmers. In this study, we used marigold, cosmos, sunflower and sesame, but other suitable ail crops such as okra and common bean also have nectar-rich flowers and could provide additional income.

## Conclusion

The highest number of natural enemies and percent parasitism of insect pest eggs by parasitoids/parasites in rice fields near nectar-rich flowering plants and the lowest number of natural enemies and parasitism were found in rice fields where insecticides were applied prophylactically. Moreover, there was no yield reduction observed in the rice field surrounded by flowering plants compared to those with insecticide applications. This suggests that farmers could avoid the use of toxic and hazardous insecticides to control insect pests by growing nectar-rich flowering plants on the bunds surrounding rice crops. This approach could help restore rice ecosystems, conserve natural enemies that ultimately help reduce production costs and chemical inputs, improve environmental protection, reduce pest infestations, and reduce labor in terms of pesticide spraying and thus increase net income. In Bangladesh, the land is fragmented into numerous patches of small size with boundaries that can be used to grow flowering plants. This creates more opportunities to use this technology in the rice landscape and the remaining challenge is to spread this technology among farmers in Bangladesh.

## Materials and Methods

### Study sites

The study was conducted at the main Bangladesh Rice Research Institute (BRRI) research facility at Gazipur (24°0′N, 90°25.5′E), and regional rice field research facility at Rajshahi (24°22′26.40″N, 88°36′4.10″E), Bangladesh. Both study sites contain rice production systems typical across the country. At each facility approximately 90% of the total area is managed for rice production and thus they are representative of the regions rice production landscapes.

### Experimental design

The experiment was conducted over two consecutive years spanning multiple rice growing seasons. Three rice growing seasons are commonly recognized in Bangladesh termed the Aus, transplanted Aman (hereafter T. Aman) and Boro seasons. The experiments described here inculded the Boro and T. Aman seasons. Three treatments were used in the study. The first treatment had flowering plant borders established on the earthen bunds (Fig. 1) with no insecticides used on the rice (hereafter T_1_). In the second treatment, the bund was left fallow and insecticides were applied to rice as per routine farming practices in Bangladesh (i.e. insecticide applied prophylactically at a 15-day intervals (T_2_). The third treatment was a control with fallow bunds and all insecticide use withheld (T_3_). The flower plant borders consisted of sunflowers (*Helianthus annus*, marigolds (*Tagetes* ssp.), and cosmos (Cosmos spp.), planted on rice bunds in the Boro season, and sesame (*Sesamum indicum*), planted on the bunds in the T. Aman rice-growing season. Sunflower and marigold were planted @ 15000 plants/ha and 30000 plants/ha respectively. Sesame was broadcasted on the bund @ 2 kg/ha. At each site, the overall experiment included 12 individual plots surrounded by bunds, arranged in 3 blocks and treatments were assigned to plots in a randomized complete block design (n = 3).

#### Gazipur

The mega rice cultivars, BRRI dhan28, BRRI dhan58 and BRRI dhan52, were cultivated in the Boro 2015–16 and T. Aman 2016 seasons, respectively. According to the respective cultivar production package, 30- to 40-day-old rice seedlings were manually transplanted into fields. Two to three seedlings were transplanted onto a hill at 20 × 20 cm spacing. All fertilizers and irrigation were applied subsequently according to treatment. The application of insecticide in T_2_ was initiated 15-days after the date of transplanting using Virtako 40WG (thiamethoxam 20% + chlorantraniliprole 20%) applied at 75 g/ha and subsequently at 15-day intervals (3 times) over the season. Bangladeshi farmers usually apply this insecticide for rice stem borer control.

#### Rajshahi

The experiment was conducted with BRRI dhan63 during the Boro 2015–16 season and with BRRI dhan52 during the T. Aman 2016 season. Nectar-rich flowering plants (cosmos in the T. Aman season and marigold in the Boro season) were planted on the bunds in each plot of T_1_. The insecticide (carbofuran 5G at 10.0 kg/ha) was applied at 15-day intervals (four times) in T_2_ after the 1^st^ top dressing of urea.

### Sampling insect pests and damage

#### Sweepnet sampling

Insects were sampled in each treatment at the maximum tillering stage of rice. A sweep net (40-cm diameter) was used to sample insect pests and natural enemies in all plots. Twenty complete sweeps were made randomly at the canopy level of the plants. The collected insects were kept in labeled bags and transported to the laboratory for sorting, identification and quantification.

#### Yellow sticky traps

Yellow sticky traps (20 × 25 cm; Zhangzhou Enjoy Agriculture Technology Co., Ltd., Fujian, China) were also used to record insect pests and their natural enemies in the experimental plots. Individual traps were fixed to bamboo canes and placed in rice plots such that sticky boards were positioned just above the plant canopy. After 48 h, all sticky traps were removed, taken to the laboratory and stored at 4 °C until all insects were counted and identified. This sticky trap was used only in one site due to lack of sufficient number of traps.

#### Parasitism of key pests

Percent parasitism of key pests was monitored using sentinels eggs of the brown planthopper (BPH), white-backed planthopper (WBPH), rice hispa and yellow stem borer (YSB). To create sentinel egg baits, potted 45-day-old rice plants were exposed to the respective pests in the lab. For the BPH and WBPH, five gravid females were caged on each pot with six plants for 48 h after which adult planthoppers were removed and the plants placed in the experimental plots. Three pots with six plants/pot each were placed in random positions centrally in each plot. For YSB, moths were captured from the field and caged on greenhouse-grown potted rice plants. After 24 h, for oviposition, YSBs were removed and plants were placed in the field in a similar manner to that used for planthopper eggs. Rice hispa egg bait traps were similarly prepared and placed in the field. After 48 h of exposure to parasitoids in the field, the plants were returned to the laboratory and kept in a greenhouse at room temperature for 4 days to allow parasitoid emergence. After this time, the plants were microscopically observed to determine the number of parasitized and nonparasitized eggs.

#### Damage assessment

The hill counting method^57^ was also used to record insect pests, their damage symptoms and the number of natural enemies per hill. During the reproductive stage of rice crops, damage symptoms, termed white head, caused by YSBs were recorded and expressed as % white head. The % white head was calculated using the formula below.

$${\rm{White}}\,{\rm{head}}\,( \% )=\frac{{\rm{No}}.{\rm{of}}\,{\rm{damaged}}\,{\rm{hills}}\,(\mathrm{of}\,{\rm{20}}\,\mathrm{hills})}{{\rm{Hills}}\,{\rm{sampled}}\,(\mathrm{20}\,\mathrm{hills})}\times \frac{{\rm{damaged}}\,{\rm{panicles}}\,(\mathrm{of}\,{\rm{20}}\,\mathrm{hills})}{{\rm{Totle}}\,{\rm{panicles}}\,({\rm{of}}\,20\,{\rm{hills}})}\times 100$$

#### Statistical analysis

The goal of this study was to evaluate the effect of flowering plants on rice bunds on pest and their natural enemies in rice fields. Treatment effects on the abundance of predators, parasitoids, insect pests, parasitism and rice yields were analyzed by one-way ANOVA, and a post hoc Tukey’s honest significance test (Tukey’s HSD). To improve homogeneity, where needed the data were log or arc sine transformed before analysis. In addition, when the value of the data was 0, then transformation was conducted by ln(n + 1). All data were analyzed using SPSS software version 16.0.

## Supplementary information

Supplementary figures and Tables
