# Supplementary material for: Establishing next-generation pest control services in rice fields: eco-agriculture
Source: Sci Rep. 2019 Jul 15;9:10180. doi: 10.1038/s41598-019-46688-6 (PMC6629669; doi:10.1038/s41598-019-46688-6)
Supplement: Supplementary file 1 — Supplementary figures and Tables [file 41598_2019_46688_MOESM1_ESM.docx]

**Supplementary information**

Establishing next-generation pest control services in rice fields: eco-agriculture

MP Ali^1*^, MN Bari^1^, SS Haque^1^, MMM Kabir^1^, S Afrin^1^, F Nowrin^1^, S Islam^2^ and DA Landis^3^

^1^Entomology Division, Bangladesh Rice Research Institute, Gazipur-1701, Bangladesh

^2^Farm Division, Bangladesh Rice Research Institute, Gazipur-1701, Bangladesh

^3^Laboratory of Entomology, Michigan State University, East Lansing, MI, USA

^*^Corresponding author email address: panna_ali@yahoo.com


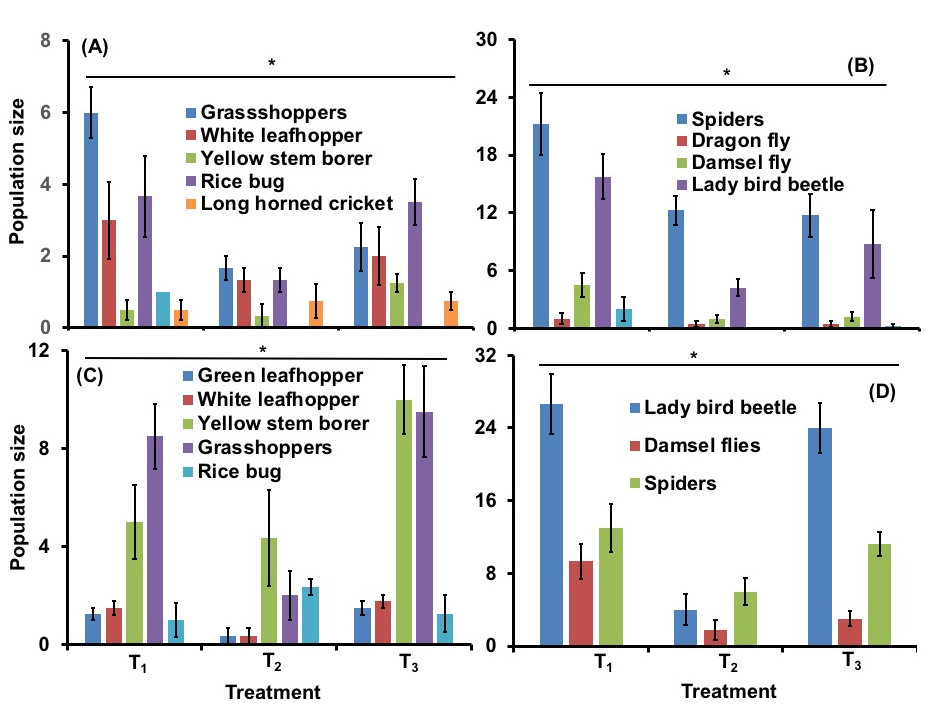


**Figure S1.** Impacts of treatments on the abundance of insect pests and natural enemies in the rice field at Rajshahi during Boro season. (A) Number of insect pest observed, Boro 2016-17; (B) Number of natural enemies observed, Boro 2016-17; (C) Number of insect pests observed, Boro 2015-16; (D) Number of natural enemies observed, Boro 2015-16. T_1_ = Flowering plants grown on rice bunds, T_2_ = Prophylactic insecticide use, and T_3_ = Control. * indicates a significant difference among treatments at the 5% level of significance. Error bar indicates standard error.


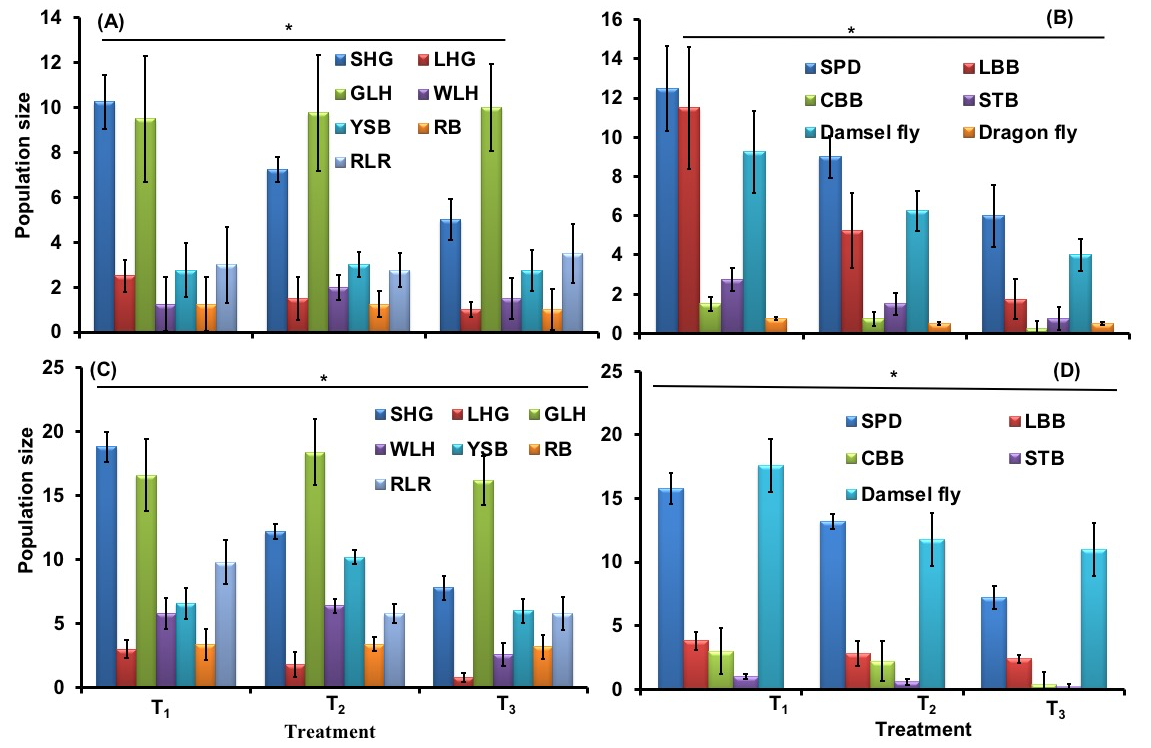


**Figure S2.** Impact of flowering plants grown in plots on the population of arthropods at Rajshahi during T. Aman season. (A) Number of insect pests observed, T. Aman 2016; (B) Number of natural enemies observed, T. Aman 2016; (C) Number of insect pests observed, T. Aman 2015; (D) Number of natural enemies observed at T. Aman 2016. T_1_ = Flowering plants grown on rice bunds, T_2_ = Prophylactic insecticide use, and T_3_ = Control. * indicates a significant difference among treatments at the 5% level of significance. Error bar indicates standard error. SHG = Short horned grasshoppers, LHG = Long horned grasshoppers, GLH = Green leafhopper, YSB = Yellow stem borer, RLR = Rice leaf roller, RB = Rice bug, WLH = White leafhopper , SPD = Spiders, CBB = Carabid beetle, LBB = Lady bird beetle, STB = Staphylinid beetle.


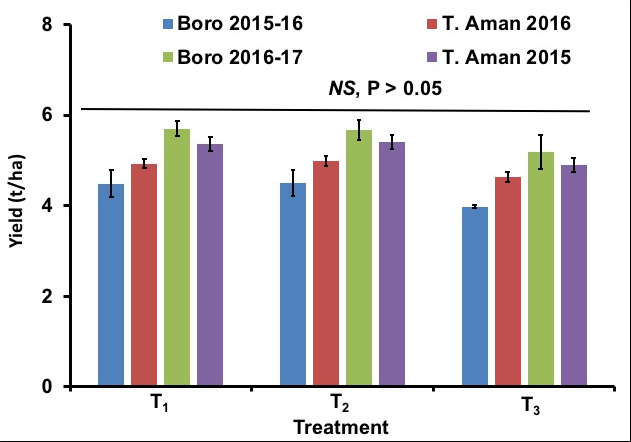


**Figure S3.** Effects of treatments on the rice yield during different years and seasons at Rajshahi. BRRI dhan52 was used during T. Aman 2015 and 2016; BRRI dhan58 was used during Boro 2015-16 and 2016-17. T_1_ = Flowering plants grown on rice bunds, T_2_ = Prophylactic insecticide use, and T_3_ = Control. *NS* indicates nonsignificant at the 5% level of significance. Error bar indicates standard error.

**Table S1.** Effect of treatment on insecticide application, yield and finally benefit cost ratio (BCR). Variety: BRRI dhan52 use in T. Aman 2016 . T_1_ = Flowers plants grown in bund around the rice plot; T_2_ = Prophylactic insecticide use; and T_3_ = Control (No insecticide and no flowering plants).

| **Treatments** | **Insecticide applied (times)** | **Insecticide reduction compared to T_2_** | **Yield (t/ha)** | **Cost for insecticide/ha**  **(Tk)** | **BCR** |
| --- | --- | --- | --- | --- | --- |
| T_1_ | - | 100% | 4.93 ± 0.09 | 00.00 | 2.19 |
| T_2_ | 04 | - | 4.99 ± 0.02 | 6000.00 | 2.00 |
| T_3_ | - | 100% | 4.64 ± 0.07 | 00.00 | 2.06 |
| *F-value* | - |  | *2.185* | - | - |
| *Significance* |  |  | *NS* |  |  |

Data were analyzed using one-way ANOVA; *NS* = not significantly different at 5% level.

**Table S2:** Effect of treatments on the benefit cost ratio (BCR) of different rice varieties cultivated at different seasons in Bangladesh. BCR was calculated based on rice production cost and return from one hectare of land.

| **SL No.** | **Season** | **Variety** | **Treatments** | **BCR** |
| --- | --- | --- | --- | --- |
| 1 | Transplanted Aman 2016  (T. Aman 2016) | BRRI dhan52 | T_1_  T_2_  T_3_ | 2.19  2.00  2.06 |
| 2 | Boro 2016-17 | BRRI dhan28 | T_1_  T_2_  T_3_ | 1.88  1.74  1.76 |
| 3 | Boro 2017-18 | BRRI dhan58 | T_1_  T_2_  T_3_ | 2.43  2.20  2.35 |
| 4 | Transplanted Aman 2017  (T. Aman 2016) | BRRI dhan52 | T_1_  T_2_  T_3_ | 1.38  1.24  1.34 |

T_1_ = Flowers plants grown in bund around the rice plot; T_2_ = Prophylactic insecticide use; and T_3_ = Control (No insecticide and no flowering plants).
